# Supplementary material for: Integrating bulk and single-cell RNA sequencing reveals SH3D21 promotes hepatocellular carcinoma progression by activating the PI3K/AKT/mTOR pathway
Source: PLoS One. 2025 Apr 3;20(4):e0302766. doi: 10.1371/journal.pone.0302766 (PMC11967960; doi:10.1371/journal.pone.0302766)

Supplementary Fig 1. Western blot analysis showed that SH3D21 protein level in samples of hepatoma patients was significantly higher than that in adjacent normal liver tissues. corresponding figure 1M.


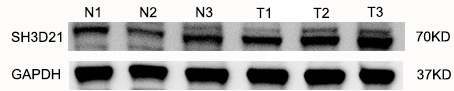


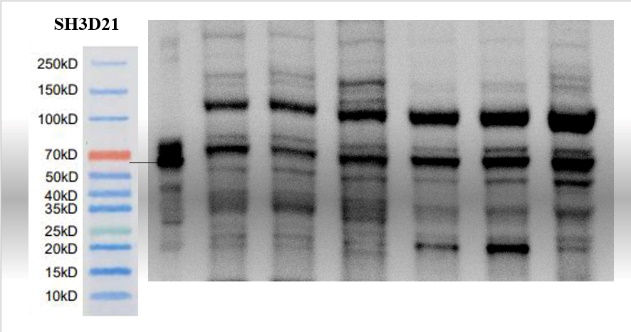

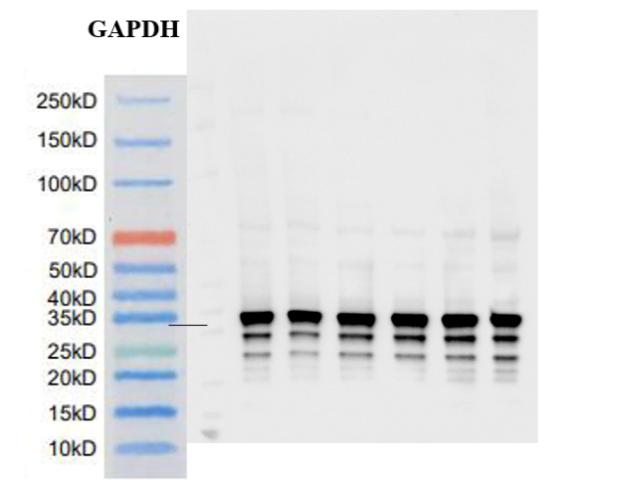


Supplementary Fig 2. Verification results revealed that SH3D21 protein expression levels in HCC cell line were markedly higher than the human hepatic stellate cells, corresponding figure 3B.


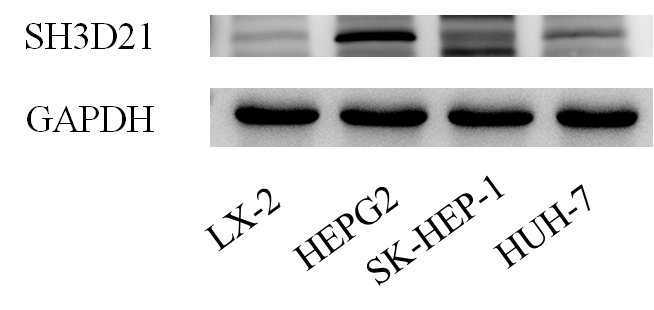

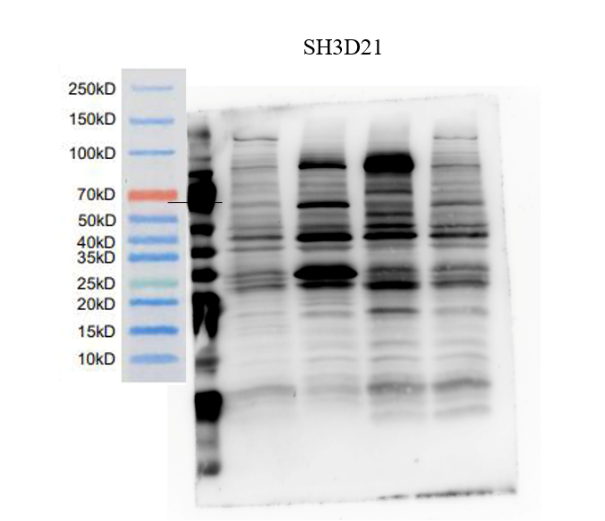

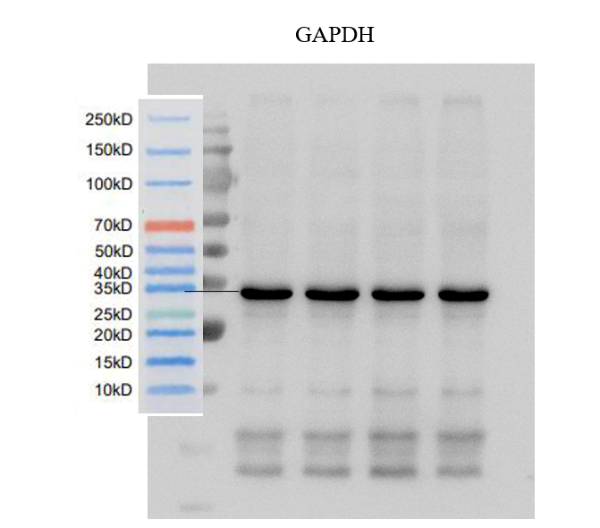


Supplementary Fig 3, The protein expression levels of SH3D21 after transfection with pcDNA3.1-SH3D21 and si-SH3D21-1, 2, 3. corresponding Fig3C-3E.


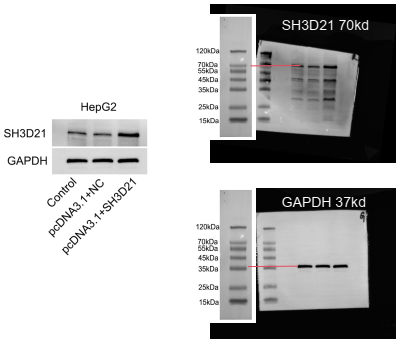


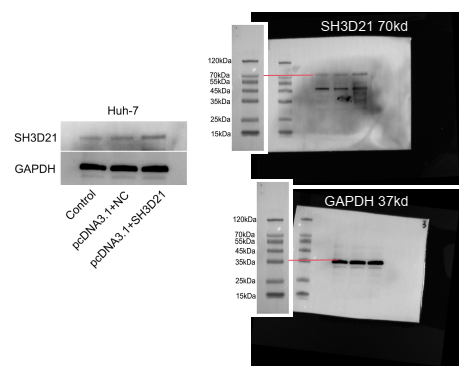


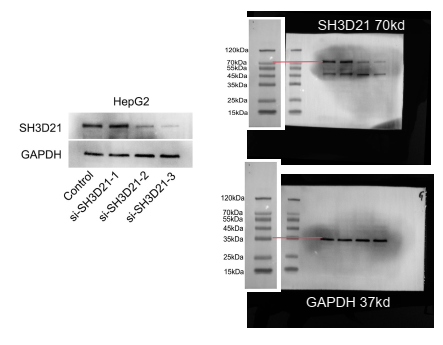


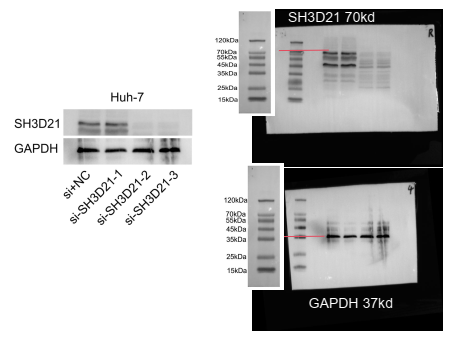


Supplementary Fig 4. In vitro in HepG2 cells experiments were performed to analyze the effect of SH3D21 on activation of PI3K/AKT signaling pathway, corresponding Fig7.


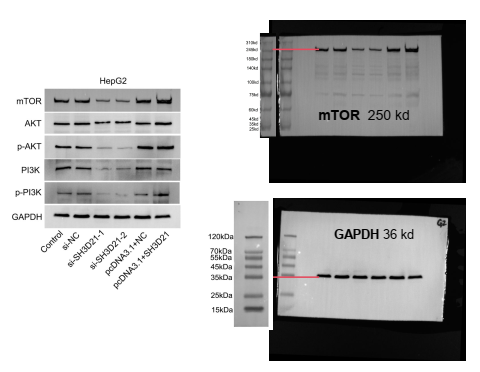


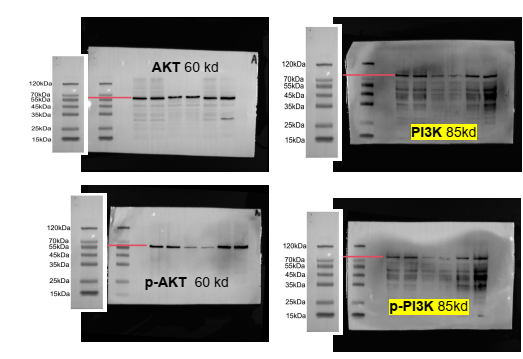


Supplementary Fig 4. In vitro in Huh-7 cells experiments were performed to analyze the effect of SH3D21 on activation of PI3K/AKT signaling pathway, corresponding Fig7.


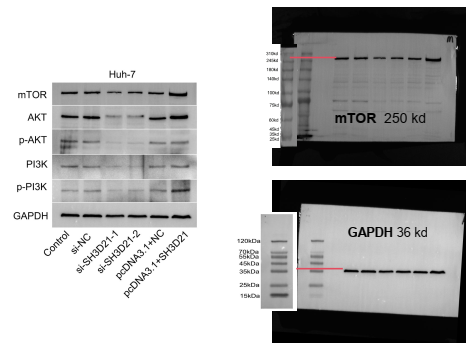


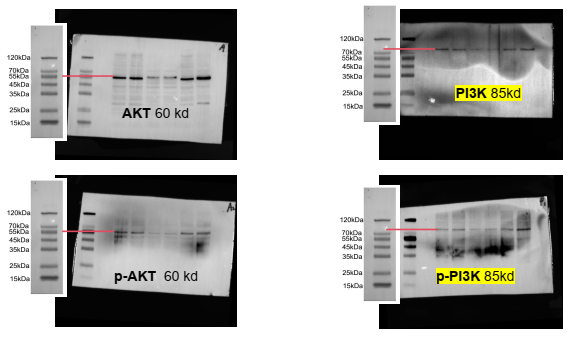

Supplement: S1 Data — (DOCX) [file pone.0302766.s001.docx]
